# Effects of nutritional stress on soil fertility and antioxidant enzymes of rice in different growth periods

**DOI:** 10.3389/fpls.2024.1471682

**Published:** 2024-10-15

**Authors:** Qiuliang Cai, Yi Zou, Ning Zhong, Guikang Jia

**Affiliations:** ^1^ Guangxi Key Laboratory of Mango Biology, School of Agriculture and Food Engineering, Baise University, College of Baise, Baise, Guangxi, China; ^2^ College of Chemistry, Chemical Engineering & Environmental Science, Minnan Normal University, Zhangzhou, Fujian, China

**Keywords:** rice, nutritional stress, enzyme activity, soil nutrients, antioxidant enzyme

## Abstract

Stress in plants denotes the detrimental impact of alterations in external environmental conditions on regular plant growth and development. Plants employ diverse mechanisms to mitigate or evade nutritional stress-induced damage. In order to investigate the physiological response mechanism of plants to nutritional stress and assess its impact on soil nutrient content and antioxidant enzyme activity in rice, a field experiment was conducted applying five treatments: control, nitrogen (N) deficiency, phosphorus (P) deficiency, potassium (K) deficiency, and full fertilization. Rice leaf and soil samples were concurrently gathered during both the vegetative and reproductive growth stages of rice. Analysis was conducted on soil N, P, and K levels, as well as leaf antioxidant enzyme activities, to investigate the impact of nutrient stress on rice antioxidant enzymes and soil fertility. The research findings indicate that full fertilization treatment enhanced the agronomic properties of the soil compared to the control treatment. In the N-deficiency treatment, reactive oxygen species (ROS) levels increased by 16.53–33.89% during the reproductive growth period compared to the vegetative growth period. The peroxidase (POD) activity decreased by 41.39% and superoxide dismutase (SOD) activity increased by 36.22% under K-deficiency treatment during the reproductive growth period compared to the vegetative growth period. Consequently, applying N and P fertilizer during the vegetative growth period can decrease membrane lipid peroxidation levels by 7.34–72.53%. The full fertilization treatment markedly enhanced rice yield compared to other treatments and increased the Nitrogen activation coefficient (NAC) and Phosphorus activation coefficient (PAC) in the soil, while decreasing the PAC. Elevating NAC levels can stimulate the activity or content of PRO, MDA, and RPS during the vegetative growth stage, whereas in the reproductive growth stage, it will decrease the content of ROS, PRO, and MDA. This data offers valuable insights and theoretical support for nutritional stress research.

## 1 Introduction

Stress in plants results from adverse alterations in external natural conditions, encompassing biotic and abiotic stressors. Biotic stress and abiotic stress collectively contribute to the challenging environmental conditions that plants face. Abiotic stress arises from variations in external natural conditions (Cheng et al., 2023; June-Sik et al., 2024; Zulfiqar et al., 2024), including cold, high temperatures, salinity, among others, leading to plant damage and impacting their regular growth and development. In contrast, biotic stress results from damage inflicted on plants by microorganisms, diseases, pests, and similar factors. Plant damage, whether direct or indirect, significantly impacts their survival and growth. To combat stress, plants have evolved diverse strategies and mechanisms (Khan and Khan, 2017; Hamideh et al., 2021), such as antioxidant systems and penetration regulation, to mitigate or prevent stress-induced damage. In the typical growth environment of plants, the equilibrium of internal free radical metabolism is maintained through elimination processes. Nevertheless, under stress conditions, cellular free radical metabolism becomes disrupted, leading to the accumulation of free radicals and reactive oxygen species in plants. As stress intensifies, plants exhibit slower growth and/or stunted stature, impacting yield quality (Ma et al., 2024). Simultaneously, stress exerts a pervasive influence on plants, disrupting the dynamic equilibrium of reactive oxygen species. Low temperature stress diminishes the activities of superoxide dismutase (SOD), catalase (CAT), and peroxidase (POD) in rice (Xu et al., 2020). Plants mitigate stress-induced damage by accumulating significant levels of proline and enhancing enzyme activity (Wang et al., 2019; Wang et al., 2021). Prior research has established that drought stress induces the accumulation of reactive oxygen species (ROS) in rice, which, when present in excess, leads to the degradation of macromolecular structures, such as nucleic acids and proteins. Concurrently, the protective enzyme system can eliminate the ROS produced during heat stress, aiding rice in adapting to high-temperature conditions (Møller et al., 2007; Ueda et al., 2013). The aforementioned study has centered on investigating the impact of salinity, alkalinity, temperature, and drought stress on rice growth, enzyme activity, and osmotic regulatory substances.

Nitrogen (N), phosphorus (P), and potassium (K) are essential elements for plant growth and represent crucial factors influencing crop nutrition. Prolonged artificial fertilization has led to soil fertility imbalances, particularly in terms of soil nutrient activation. Nutrient activation denotes the comprehensive impact of soil nutrient efficacy on soil properties, plant growth, nutrient varieties, and related factors. Nonetheless, the prevalence of soil fertility disparities has become a prevalent occurrence in cultivated areas, underscoring the significance of investigating nutritional stress. Nutritional stress encompasses the plant growth responses to conditions of nutrient deficiency or excess. Adverse environments disrupt the typical growth parameters of plants and induce alterations in their biological functions. Stress related to N, P, and K arises from artificially subjecting plants to unfavorable growth conditions. Hence, there remains a need to augment the existing literature concerning the impact of nutritional stress on crop antioxidant enzyme activity and soil fertility.

Oryza sativa, a key staple crop globally (Hu. et al., 2020), holds significant importance in agricultural production (Ye et al., 2012). Nonetheless, extensive use of chemical fertilizers has led to nutrient depletion in cultivated lands. Imbalanced soil fertility may trigger nutrient stress during rice cultivation. Addressing this gap, the present study focuses on rice, a predominant food crop, as the research subject, with five treatments (control, nitrogen (N) deficiency, phosphorus (P) deficiency, potassium (K) deficiency, and full fertilization) administered at various growth stages of rice. Analyses were conducted on alterations in leaf enzyme activity, physiological markers, and soil nutrient levels, investigating their influence on soil nutrient and physiological responses to nitrogen (N) deficiency, phosphorus (P) deficiency, potassium (K) deficiency stress. The aim was to elucidate the physiological response mechanism of plants under nutritional stress. This study furnishes data support and a scientific foundation for understanding the mechanism of rice nutrition stress.

## 2 Materials and methods

### 2.1 Overview of the test site

Tianyang District is situated in the western region of Guangxi, within the Youjiang River Valley at coordinates 106°91′ E, 23°73′ N. The area experiences a southern subtropical monsoon climate characterized by a high average temperature, ample sunlight, plentiful rainfall, an extended frost-free period, and conducive conditions for year-round cultivation. The average annual temperature is 22°C, with an annual precipitation of 1100 mm. Heat peaks occur thrice annually in this region.

### 2.2 Test materials

#### 2.2.1 Test design, sample collection, and processing

Rice cultivation took place in Baiyu Town, Tianyang District, within a field featuring yellow mud paddy soil characterized by a clay texture. The experimental plots were level. The soil composition comprised 32.5 g/kg of organic matter, 1.705 g/kg of total nitrogen (TN), 15.1 mg/kg of available P, and 215 mg/kg of rapidly available K, with a pH level of 6.0. The rice variety utilized in the study was Liangxiang Fasi. A total of 15 plots were randomly distributed into groups. The N fertilizer employed was urea with a minimum N content of 46.2%; the P fertilizer utilized was calcium magnesium phosphate with a minimum P_2_O_5_ content of 18%; and the K fertilizer applied was K chloride with a minimum K_2_O content of 60%. According to the target yield method, the fertilizer quantities applied were determined as 150 kg N/hectare, 52.5 kg P_2_O_5_/hectare, and 135 kg K_2_O/hectare. At the same time, in order to study nutritional stress, no nitrogen, phosphorus and potassium fertilizers were applied. The detailed fertilization schedule (five treatments: control, nitrogen (N) deficiency, phosphorus (P) deficiency, potassium (K) deficiency, and full fertilization) is presented in **Table 1**.

**Table 1:** Design of fertilizer scheme.

Plot	Fertilization program	Urea (kg/hectare)	Calcium, magnesium, and phosphate fertilizers (kg/hectare)	Potassium chloride (kg/hectare)
1	Control (ck)	0	0	0
2	Nitrogen-free zone (N0PK)	0	19.44	15.00
3	Phosphorus-free zone (NP0K)	21.64	0	15.00
4	Potassium-free zone (NPK0)	21.64	19.44	0
5	Nitrogen, phosphorus, and potassium area (NPK)	21.64	19.44	15.00

At approximately 40 days after planting during the rice vegetative growth stage and around 90 days after planting during the reproductive growth phase, 10 leaves were harvested from each plot. These collected leaves were combined in sample bags, labeled, and transported to the laboratory for initial grinding and freezing pretreatment. A five-point sampling technique utilizing the double diagonal method was employed to gather soil samples (from 0 to 20 cm depth) from each plot. Each soil sample from a plot weighed approximately 500 g and was placed in sample bags for transportation back to the laboratory. Following the removal of debris, the soil samples underwent natural air drying before being ground through a 100-mesh sieve. Employing a four-point method, the soils were divided into two portions: one for soil nutrient analysis and the other for dry preservation.

### 2.3 Determination of indicators

#### 2.3.1 Determination of soil physical and chemical properties 

TN was analyzed employing the Kjeldahl distillation method, while total phosphorus (TP) and total potassium (TK) were assessed using the HClO_4_-H_2_SO_4_ digestion–molybdenum anti-coloration method (Bao, 2000). Ammonium nitrogen (NH^4+^-N) levels were determined utilizing the Nather reagent photometric method (Bao, 2000). Nitrate nitrogen (NO^3–^N) levels were quantified through ultraviolet spectrophotometry, while quick-acting phosphorus (AP) was analyzed using molybdenum antimony spectrophotometry (Bao, 2000). Soil quick-acting potassium (AK) was measured via 1 mol/L ammonium acetate extract and flame photometry (Bao, 2000).

#### 2.3.2 Determination of the physiological and biochemical indicators of the rice leaves

ROS were quantified utilizing the TMB colorimetric method as per the ELISA kit instructions. POD activity was assessed employing the oxidyl method, while CAT activity was measured via ultraviolet spectrophotometry (Li, 2000). SOD activity was quantified utilizing the enzyme-meter colorimetric method. The proline content was assessed employing the acidic indotriene method (Zhu et al., 1983). Membrane lipid peroxidation, indicated by malondialdehyde levels, was determined using the thiobarbituric acid (TBA) colorimetric assay (Heath and Packer, 2022). Chlorophyll concentration was measured through the 96% ethanol extraction method (Zhao and Cang, 2016).

### 2.4 Data processing


$$\begin{array}{c} \text{Nitrogen activation coefficient (NAC)}\\ =(\text{concentration of }{\text{NH}}_{+}^{4}-\text{N}\\ +{\text{concentration of NO}}^{3-}-\text{N})/\text{concentration of TN} \end{array}$$

$$\begin{array}{c} \text{Phosphorus activation coefficient }\left(\text{PAC}\right)\\ =\text{concentration of AP}/\text{concentration of TP} \end{array}$$

$$\begin{array}{c} \text{Potassium activation coefficient }\left(\text{KAC}\right)\\ =\text{concentration of AK}/\text{concentration of TK} \end{array}$$

The experimental data were initially organized using Microsoft Excel 2020 software. IBM SPSS Statistics 22 software was employed for variance analysis, multiple comparisons, Pearson correlation analysis, and principal component analysis. Redundancy analysis was conducted using Canoco 5 software, and graphical representations were generated using Origin 2022.

## 3 Results

### 3.1 Effects of nutritional stress on the agronomic characteristics of rice

As illustrated in **Table 2**, the plant height reached its peak (123.00 cm) with the NPK fertilization treatment and was lowest in the control group. N deficiency treatment exhibited a substantial influence on the number of effective panicles per hectare. The highest number of effective panicles (14.92; tens of thousands) was observed in the complete fertilization treatment, while the lowest (11.38; tens of thousands) was recorded in the control group. Additionally, the grain-to-panicle ratio was notably lower in the complete fertilization treatment compared to the other experimental treatments. The grain yield in the complete fertilizer treatment reached 7680 kg/hectare, exhibiting a 25.60% increase compared to the control group. Furthermore, the highest straw yield was recorded in the complete fertilizer treatment, reaching 11098.20 kg/hectare, signifying a 32.43% increase relative to the control group.

**Table 2:** Agronomic characteristics of rice in differently fertilized treatments.

Plot	Straw height (cm)	Effective panicles per acre (tens of thousands)	1000-grain weight (g)	Valley/pole	Grain yield (kilograms per hectare)	Straw yield (kilograms per hectare)
1	116.33 ± 3.09b	11.38 ± 0.49c	22.32 ± 1.139a	0.730 ± 0.035b	6114.60 ± 305.73b	8376.15 ± 418.80b
2	117.67 ± 1.70b	12.35 ± 1.48ab	21.65 ± 1.083a	0.752 ± 0.038b	7157.55 ± 357.89ab	9517.95 ± 475.95ab
3	120.33 ± 3.09ab	13.26 ± 3.12b	22.51 ± 1.126a	0.740 ± 0.037b	7317.604 ± 365.88ab	9888.60 ± 494.40ab
4	119.67 ± 1.25ab	14.01 ± 0.93a	22.47 ± 1.124a	0.762 ± 0.038b	7654.80 ± 382.74a	10045.65 ± 502.35a
5	123.00 ± 0.82a	14.92 ± 2.80a	22.78 ± 1.116a	0.693 ± 0.037a	7680.00 ± 384.00a	11098.20 ± 554.85a

Different lowercase letters in the same row represent significant differences (*P*< 0.05).

### 3.2 Effects of nutritional stress on the physical and chemical properties of rice soil

#### 3.2.1 Effects of nutritional stress on the total nutrients of rice soil

**Figure 1:**
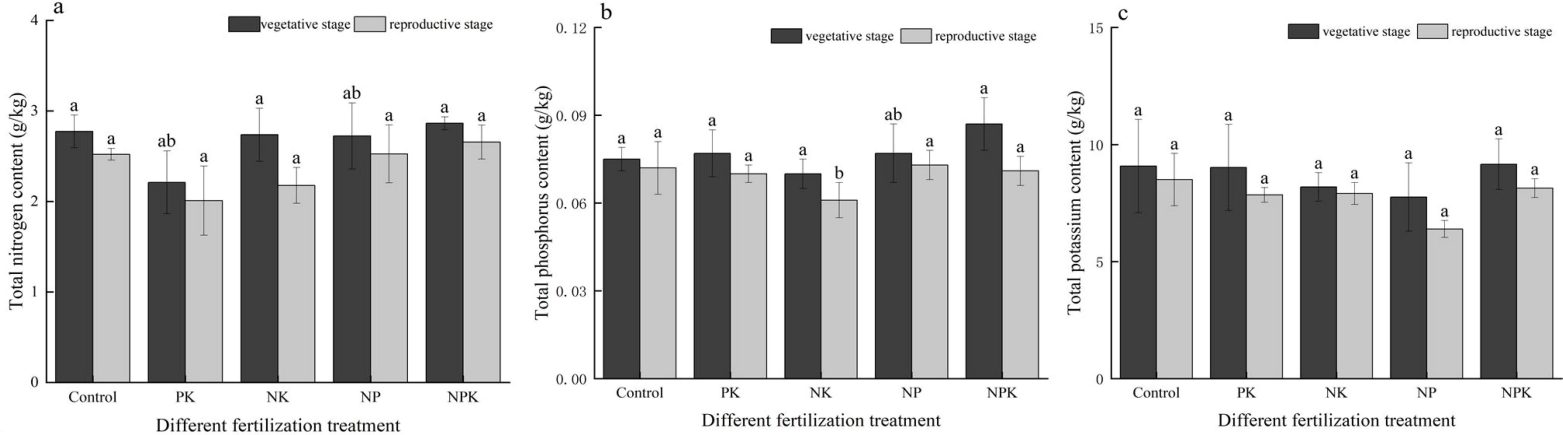
Nutrient levels of the soil under nutritional stress: total nitrogen **(A)**; total phosphorus **(B)**; and total potassium **(C)**. Different lowercase letters indicate significant differences among different fertilization treatments (P< 0.05).

As illustrated in **Figure 1**, the impacts of various fertilization treatments on soil TN, TP, and TK contents varied. The levels of TN, TP, and TK in the soil during the reproductive growth phase were observed to be lower compared to those during the vegetative growth phase. Overall, the soil N, P, and K contents showed a decreasing trend as the rice plants progressed in growth stages. With the exception of the K deficiency treatment, the TN contents in the soil for the other treatments during the reproductive growth phase decreased by 10.00%, 10.05%, 25.67%, 7.80%, and 7.22%, respectively, in comparison to the vegetative growth phase. The largest variation in TN content between the two periods was observed with the P deficiency treatment compared to the other treatments. Additionally, the TP content of the soil during the reproductive growth phase decreased by 4.20% to 22.54% in comparison to the vegetative growth phase. The most substantial decrease (22.54%) in TP content was observed in the full fertilization treatment. Furthermore, the soil TK content during the reproductive growth phase decreased by 3.54% to 21.36% compared to the vegetative growth phase. Specifically, the soil TK content in the K-deficient treatment decreased from 7.76 g/kg to 6.394 g/kg, representing the largest decline of 21.36%.

**Figure 2:**
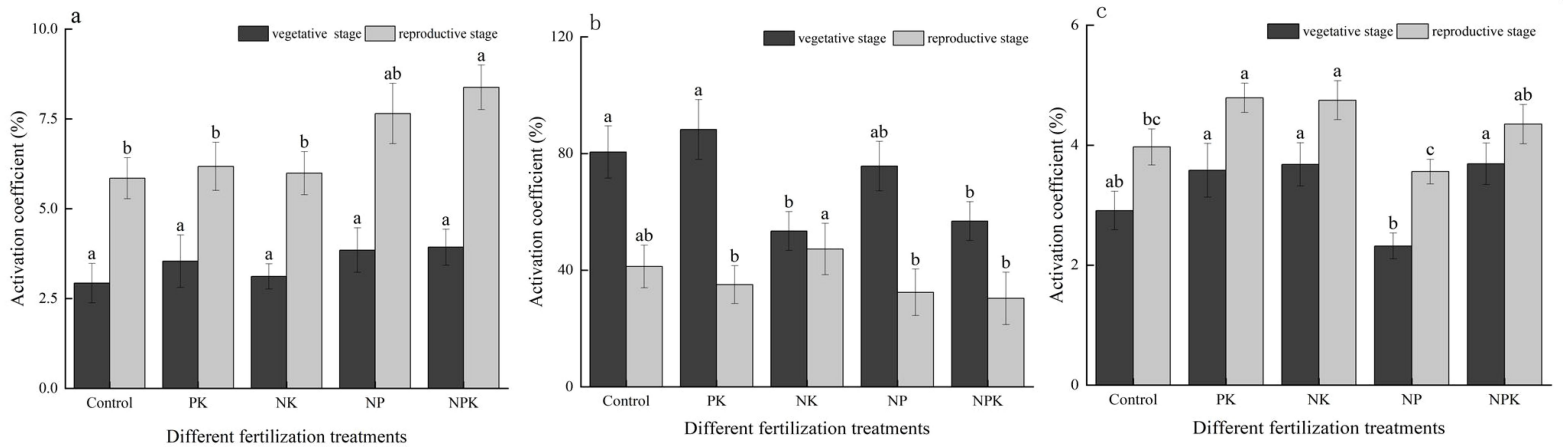
Activation coefficients of rice under nutritional stress: nitrogen **(A)**; phosphorus **(B)**; and potassium **(C)**. Different lowercase letters indicate significant differences among different fertilization treatments (P< 0.05).

As depicted in **Figure 2**, fertilization notably enhanced the soil nutrient activation coefficient for N, P, and K. Specifically, during the vegetative growth phase of rice, the activation coefficients for N, P, and K nutrients across various fertilization treatments ranged from 2.93% to 3.93%, 53.45% to 80.50%, and 2.32% to 3.69%, respectively. In the reproductive growth stage of rice, these values were observed to be 5.85% to 8.38%, 30.35% to 47.25%, and 3.56% to 4.79% for N, P, and K nutrients, respectively. In comparison to the full fertilization treatment during the vegetative and reproductive growth stages, the N activation coefficients decreased by 11.02% and 35.60% in the N deficiency treatment, the P activation coefficients decreased by 6.34% and 55.68% in the P deficiency treatment, and the K activation coefficients decreased by 59.05% and 22.19% in the K deficiency treatment, respectively. Significant variations were observed in the activation coefficients of P and K nutrients among the treatments during the vegetative growth stage. Additionally, the activation coefficients of N, P, and K displayed significant differences among the treatments during the reproductive growth stage of rice.

#### 3.2.2 Effects of nutritional stress on nutrient absorption of rice and fertilizer use efficiency

As indicated in **Table 3**, N absorption decreased by 18.30 kg/hectare with the N deficiency treatment compared to the full fertilization treatment, and increased by 0.41 kg/m² compared to the blank treatment. The total nutrient absorption in the P deficiency treatment did not exhibit a significant difference compared to that in the blank treatment. Nevertheless, it was 14.5% lower than that observed under the full fertilization treatment. The TK absorption varied by 1.30 kg/m² between the K deficiency treatment and the blank treatment. Notably, with the full fertilization treatment, the TK absorption was 3.17 kg/m² higher than that recorded for the K deficiency treatment. The fertilizer utilization rates were ranked as follows: K fertilizer (35.22%) > P fertilizer (32.86%) > N fertilizer (12.14%). The utilization rate of N fertilizer throughout the season was notably low at 12.14%. Furthermore, the total amounts of N, P, and K absorbed by the crops exhibited variations.

**Table 3:** Absorption and fertilizer utilization rates of different fertilizer nutrients.

Plot	Total nutrient uptake (kg/hectare)			Fertilizer utilization efficiency (%)		
	N	P_2_O_5_	K_2_O	Nitrogenous fertilizer	Phosphate fertilizer	Potassium fertilizer
1	18.15	115.50	129.60			
2	24.30			12.14		
3		118.95			32.86	
4			149.10			35.22
5	42.60	136.20	196.65			

### 3.3 Effects of nutritional stress on the physiological and biochemical indicators of rice leaves

#### 3.3.1 Changes in antioxidant enzyme activities (POD, CAT, and SOD) in rice leaves under nutrient stress

**Figure 3:**
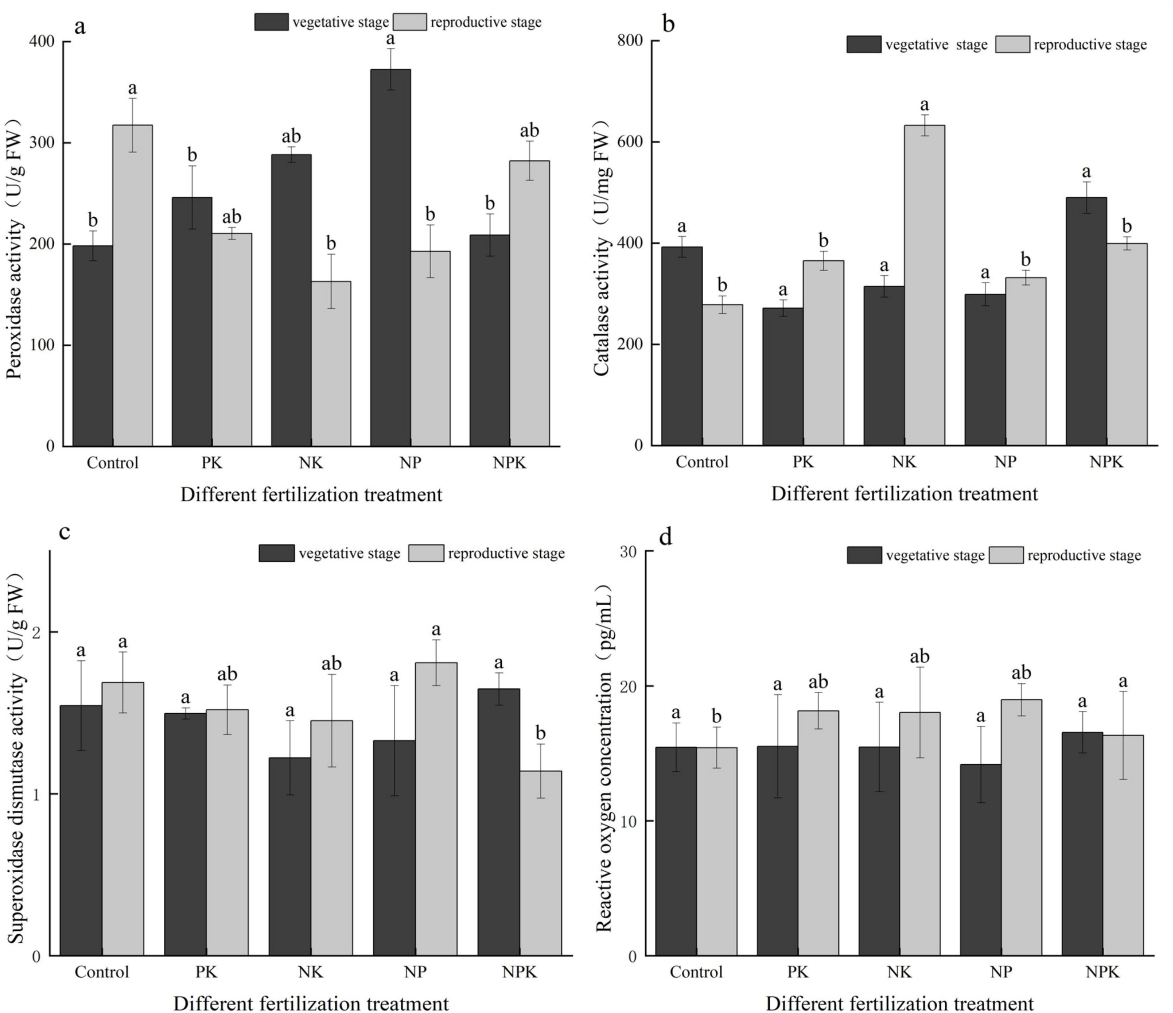
Activities of the antioxidant enzymes. **(A)** POD; **(B)** CAT; **(C)** SOD; and **(D)** ROS. Different lowercase letters indicate significant differences among different fertilization treatments (P < 0.05).

As depicted in **Figure 3**, during the vegetative growth stage, the POD activities in the N, P, and K deficiency treatments were recorded at 30,000 U/g, 35,166.67 U/g, and 45,433.33 U/g, respectively. These values were 17.80%, 38.09%, and 78.40% higher, respectively, compared to those of the full fertilization treatment. Nonetheless, the CAT activity under the three deficiency treatments exhibited a sequential decrease of 80.49%, 55.77%, and 63.96%, respectively, in comparison to the full fertilization treatment. Interestingly, the activities of POD and CAT in the mature stage of rice displayed an opposite trend to those observed in the vegetative growth stage. In the mature stage, the POD activities of the three deficiency treatments decreased by 34.09%, 32.46%, and 46.37%, respectively, compared to the full fertilization treatment. Interestingly, the CAT activities of the N and P deficiency treatments increased by 9.38% and 20.39%, respectively, when compared to the full fertilization treatment. During the rice reproductive growth stage, the POD activities of both the full fertilization treatment and the blank treatment were notably elevated compared to the vegetative growth stage, with increments of 84.68% and 118.89%, respectively. In contrast, the CAT activities of the full fertilization treatment and the blank treatment decreased by approximately 3 to 4 times from the vegetative growth stage to the reproductive growth stage.

As illustrated in **Figure 3C**, apart from the full fertilization treatment, the SOD activities of the other treatments were lower during the vegetative growth period compared to the mature period. Specifically, in the case of the K deficiency treatment, the SOD activity increased from 1.328 U/g (vegetative growth period) to 1.809 U/g (mature period), representing a rise of 36.22%. Conversely, for the full fertilization treatment, the SOD activity decreased from 1.647 U/g in the vegetative growth period to 1.14 U/g in the mature growth stage, indicating a decline of 44.47%.

As depicted in **Figure 3D**, the ROS concentrations in the N, P, and K deficiency treatments showed increments of 16.97%, 16.53%, and 33.89%, respectively, between the two periods. In contrast, the ROS concentrations in the blank treatment and the full fertilization treatment did not exhibit significant changes between the two periods.

Fertilization notably enhanced the alterations in POD activity (increase) and CAT activity (decrease) between the two periods. It was observed that the changes in SOD activity were relatively slower compared to the variations in POD and CAT activities during the two periods.

#### 3.3.2 Changes in chlorophyll content under nutritional stress

**Figure 4:**
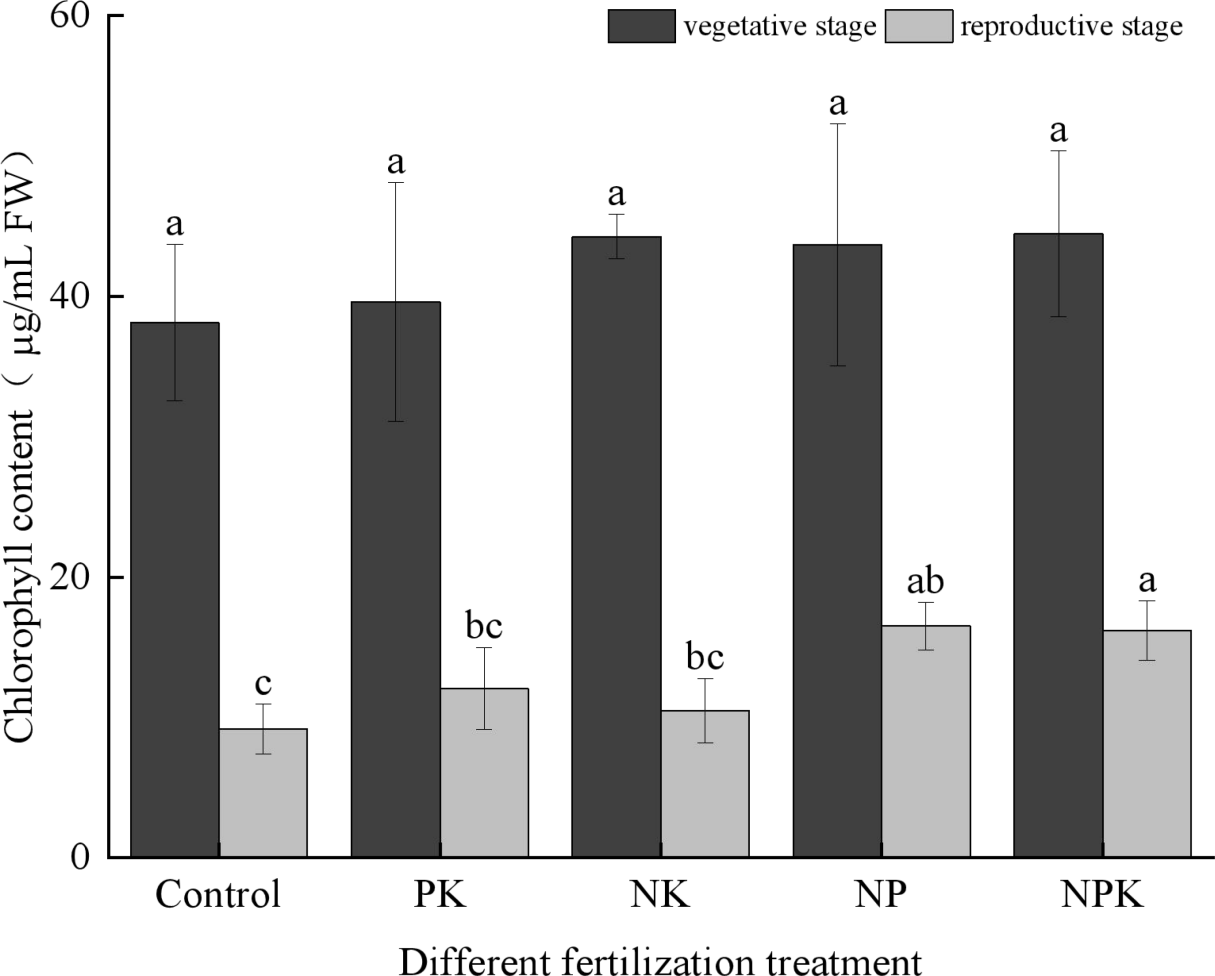
Chlorophyll contents of rice leaves under nutritional stress. Different lowercase letters indicate significant differences among different fertilization treatments (P < 0.05).

As depicted in **Figure 4**, the chlorophyll content in the rice did not exhibit significant differences among the treatments during the vegetative growth period, with levels averaging around 40 µg/mL. However, during the reproductive growth stage of the rice, significant variations in chlorophyll content were observed between the full fertilization treatment and the blank treatment, as well as the N deficiency and P deficiency treatments. The K deficiency treatment exhibited the highest chlorophyll content, measuring 16.501 µg/mL among the treatments. Interestingly, the chlorophyll content under the K deficiency treatment was comparable to that of the full fertilization treatment. The ranking of chlorophyll contents was as follows: the P deficiency treatment was approximately on par with the full fertilization treatment, followed by the N deficiency treatment, the P deficiency treatment, and finally, the blank treatment.

#### 3.3.3 Changes in the contents of osmotic and regulated substances in rice leaves under nutritional stress

**Figure 5:**
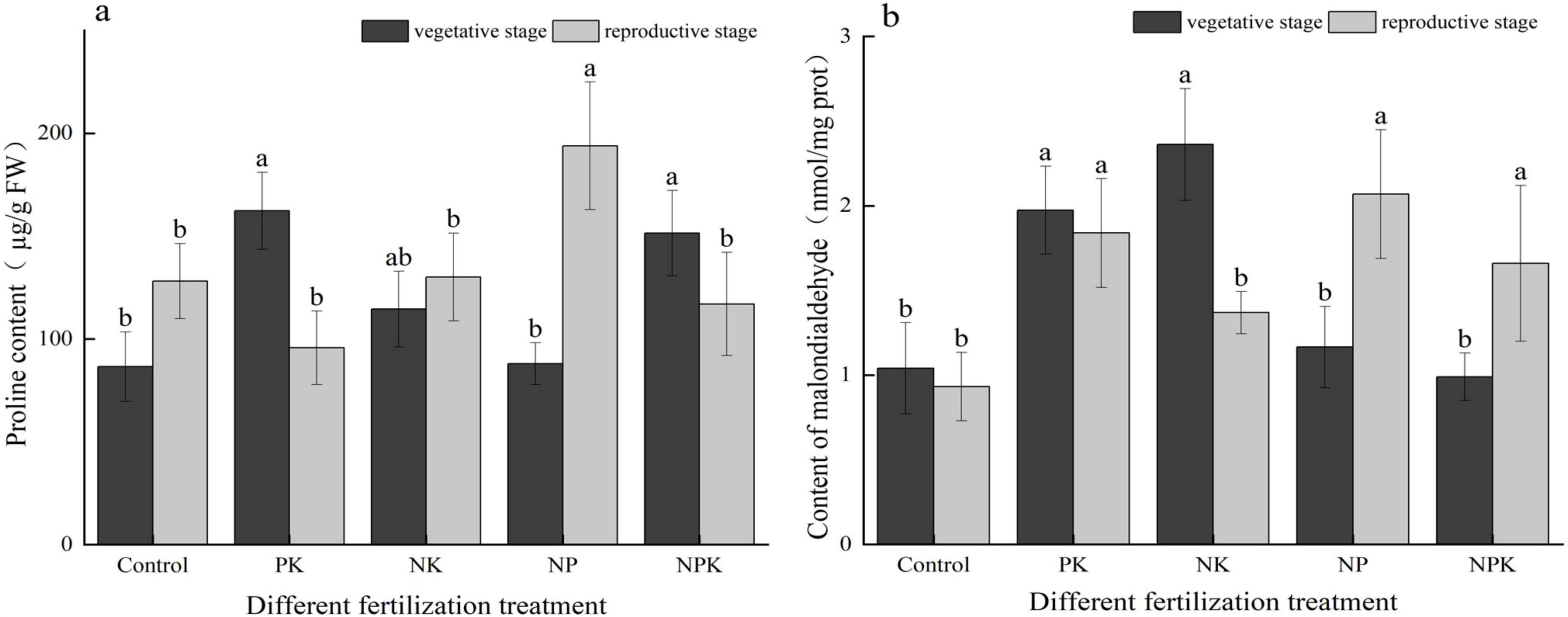
The contents of permeability regulators of rice leaves under nutritional stress **(A)** PRO; and **(B)** MDA. Different lowercase letters indicate significant differences among different fertilization treatments (P < 0.05).

As illustrated in **Figure 5A**, no significant difference was observed in the proline concentration between the N deficiency treatment and the full fertilization treatment during the vegetative growth period. Notably, the proline content in the N deficiency treatment, measuring 162.315 ug/g, was the highest among all the treatments. In comparison to full fertilization, the proline concentrations in the P deficiency and K deficiency treatments decreased by 32.23% and 72.19%, respectively. Notably, the proline content of the K deficiency treatment was the highest during the reproductive growth stage, reaching 193.966 µg/g, demonstrating a significant increase of 65.72% compared to the full fertilization treatment. The proline content of the N deficiency treatment exhibited a substantial change between the two periods, decreasing from 162.315 µg/g to 95.715 µg/g, representing a decrease of 69.58%. In contrast, the proline content of the K deficiency treatment showed an opposite trend, increasing from 89.984 µg/g to 193.966 µg/g, indicating a 1.16-fold increase.

As illustrated in **Figure 5B**, fertilization had a significant effect in reducing the MDA content of the plants. During the vegetative growth period, the MDA contents of the three nutrient deficiency treatments were all higher than those of the full fertilization treatment. Specifically, the MDA contents of the three nutrient deficiency treatments increased by 17.77% to 137% compared to the full fertilization treatment. The primary elements responsible for inducing plant membrane lipid peroxidation, leading to a reduction of MDA, were found to be P and N > K during the vegetative growth period. At the reproductive growth stage, the malondialdehyde contents in the N deficiency and K deficiency treatments were measured at 1.839 nmol/mg and 2.069 nmol/mg, respectively. These values were 10.78% and 24.64% higher than those recorded under the full fertilization treatment. The MDA content in the P deficiency treatment decreased by 72.53% during the vegetative growth period compared to the mature period. In contrast, the MDA contents in the K deficiency treatment and full fertilization treatments increased by 77.44% and 67.68%, respectively, during the same period.

### 3.4 Soil physicochemical properties under nutritional stress using plant physiology and biochemical indicators

The results of the principal component analysis conducted during the vegetative growth period are presented in **Table 4**. Based on the extraction of eigenvalues greater than 1 for the principal components, a total of five principal components were extracted. The contribution rates of the five principal components were 17.883%, 16.413%, 14.114%, 11.483%, and 9.303%, respectively, resulting in a cumulative contribution rate of 69.195%. Principal component 1 was influenced by soil nitrate N, plant catalase, and chlorophyll; principal component 2 was influenced by soil TP and TN; principal component 3 was impacted by soil available K; principal component 4 was influenced by soil ammonium N and available P; and principal component 5 was affected by plant catalase and malondialdehyde. The results of the principal component analysis during the reproductive growth stage are presented in **Table 5**. Following the extraction of eigenvalues greater than 1 for the principal components, a total of five principal components were extracted for this period. The contribution rates for these components were 20.289%, 18.620%, 14.042%, 12.361%, and 9.175%, resulting in a cumulative contribution rate of 74.487%. Principal component 1 was influenced by soil TP, total K, and plant catalase; principal component 2 was influenced by soil nitrate N and plant proline; principal component 3 was impacted by soil ammonium N, available P, and available K; principal component 4 was influenced by plant malondialdehyde and chlorophyll; and principal component 5 was affected by soil TN and plant peroxidase. The factors influencing the principal components extracted in the two periods varied, with the cumulative contribution rate in the reproductive growth period being higher than that in the vegetative growth period.

**Table 4:** The main components of the soil physicochemical properties and plant physiology and biochemical indicators under nutritional stress in the vegetative growth period.

Component	Main ingredient 1	Main ingredient 2	Main ingredient 3	Main ingredient 4	Main ingredient 5
Ammonium nitrogen	-0.170	-0.050	-0.125	-0.732	-0.073
Nitrate nitrogen	0.607	0.297	-0.114	0.192	-0.001
Available phosphorus	-0.196	-0.016	0.106	0.669	-0.015
Total phosphorus	0.078	0.872	0.051	-0.122	-0.116
Total potassium	0.082	0.727	-0.138	0.238	0.017
Total nitrogen	0.480	-0.543	-0.143	0.363	-0.126
Fast-acting potassium	-0.106	-0.058	0.779	0.110	0.037
Peroxidase	0.077	-0.228	-0.293	-0.103	0.870
Catalase	0.800	-0.073	0.082	-0.097	-0.032
Malondialdehyde	-0.254	0.306	0.308	0.397	0.694
Chlorophyll	0.661	0.025	0.321	-0.457	-0.010
Proline	-0.264	-0.014	-0.808	-0.097	0.181
Eigenvalue	2.146	1.970	1.694	1.378	1.116
Contribution rate %	17.883	16.413	14.114	11.483	9.303
Cumulative contribution rate %	17.883	34.295	48.409	59.892	69.195

**Table 5:** The main components of the soil physicochemical properties and plant physiological and biochemical indicators under nutritional stress in the reproductive growth period.

Component	Main ingredient 1	Main ingredient 2	Main ingredient 3	Main ingredient 4	Main ingredient 5
Ammonium nitrogen	-0.094	0.264	-0.871	-0.033	-0.024
Nitrate nitrogen	0.016	-0.901	0.031	0.029	0.063
Available phosphorus	0.041	0.334	0.747	0.131	0.037
Total phosphorus	0.807	-0.252	-0.130	-0.324	-0.045
Total potassium	0.808	0.037	0.047	0.195	-0.155
Total nitrogen	-0.256	-0.014	0.164	-0.273	0.736
Fast-acting potassium	-0.100	0.133	0.674	-0.116	0.181
Peroxidase	0.184	0.098	0.074	0.364	0.731
Catalase	-0.687	-0.005	-0.124	0.272	-0.455
Malondialdehyde	0.017	0.242	0.001	0.821	0.004
Chlorophyll	0.287	0.394	-0.016	-0.773	0.025
Proline	-0.127	0.814	0.189	0.079	0.149
Eigenvalue	2.435	2.234	1.685	1.483	1.101
Contribution rate %	20.289	18.620	14.042	12.361	9.175
Cumulative contribution rate %	20.289	38.909	52.951	65.312	74.487

The scores of the five principal components and the overall scores of different treatments during the rice vegetative growth stage are presented in **Table 6**. The comprehensive scores of the 12 indicators for each treatment were computed using F=0.258*F1 + 0.237*F2 + 0.204*F3 + 0.166*F4 + 0.134*F5. The ranking of the scoring results is as follows: the score for full fertilization was higher than that of the N deficiency treatment, followed by the K deficiency treatment, the P deficiency treatment, and finally, the blank treatment. The comprehensive scores of the five principal components for the various treatments at reproductive growth are presented in **Table 6**. The comprehensive scores of the 12 indicators of each treatment were calculated using F=0.272*F1 + 0.250*F2 + 0.189*F3 + 0.166*F4 + 0.123*F5, and the order of the results is as follows: the score for the N deficiency treatment was higher than that of full fertilization, followed by the blank treatment, P deficiency, and K deficiency. The highest comprehensive scores were observed in the N deficiency treatment during the reproductive growth stage and the full fertilization treatment during the vegetative growth stage.

**Table 6:** Components and comprehensive scores among treatments in the vegetative growth period and the reproductive growth period.

Research Phase	Treatment	F1	F2	F3	F4	F5	F	Rank
vegetative growth period	Blank processing	0.318	0.223	-0.212	0.397	0.698	0.186	5
	Nitrogen deficiency treatment	-0.127	0.840	0.218	0.082	1.010	0.393	2
	Phosphorus deficiency treatment	0.153	0.269	0.039	-0.026	1.056	0.209	4
	Potassium deficiency treatment	-0.131	0.904	-0.190	-0.199	1.327	0.321	3
	Full fertilization	-0.183	1.065	0.428	-0.358	1.053	0.422	1
reproductive growth period	Blank processing	0.199	0.350	0.230	1.689	-0.798	0.367	3
	Nitrogen deficiency treatment	0.478	0.518	0.332	1.106	-0.502	0.444	1
	Phosphorus deficiency treatment	0.513	0.144	0.328	1.377	-0.918	0.353	4
	Potassium deficiency treatment	-0.162	0.373	0.491	1.492	-0.751	0.297	5

### 3.5 The correlation between soil physicochemical properties and plant physiological and biochemical indicators under nutritional stress

**Figure 6:**
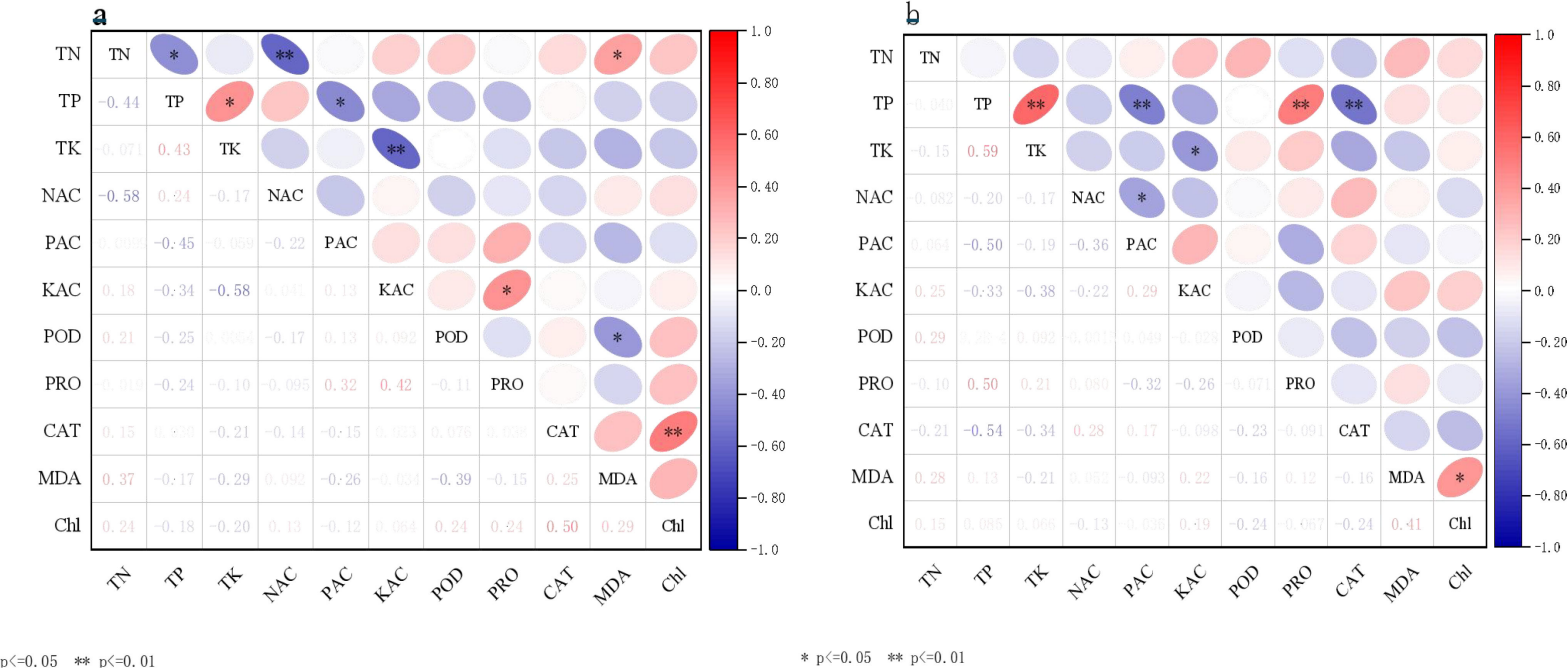
Correlation analysis between soil physicochemical properties and plant physiological and biochemical indicators. **(A)** vegetative growth period; and **(B)** reproductive growth period.

In **Figure 6A**, an exceptionally significant positive correlation was observed between chlorophyll and CAT (P< 0.01). Additionally, significant positive correlations were found between TP and TK, available K and proline, ammonium N and MDA, as well as TN and MDA (P< 0.05). Furthermore, a significant negative correlation was noted between POD and MDA (P< 0.05). No significant differences were observed among the other indicators. In **Figure 6B**, significant positive correlations were observed between TP and TK, as well as between TP and PRO. A significant negative correlation was identified between TP and CAT (P< 0.05), while a highly significant positive correlation was found between nitrate N and PRO (P< 0.01). Additionally, a significant positive correlation was noted between soil TK and TP across the two periods (P< 0.05).

**Figure 7:**
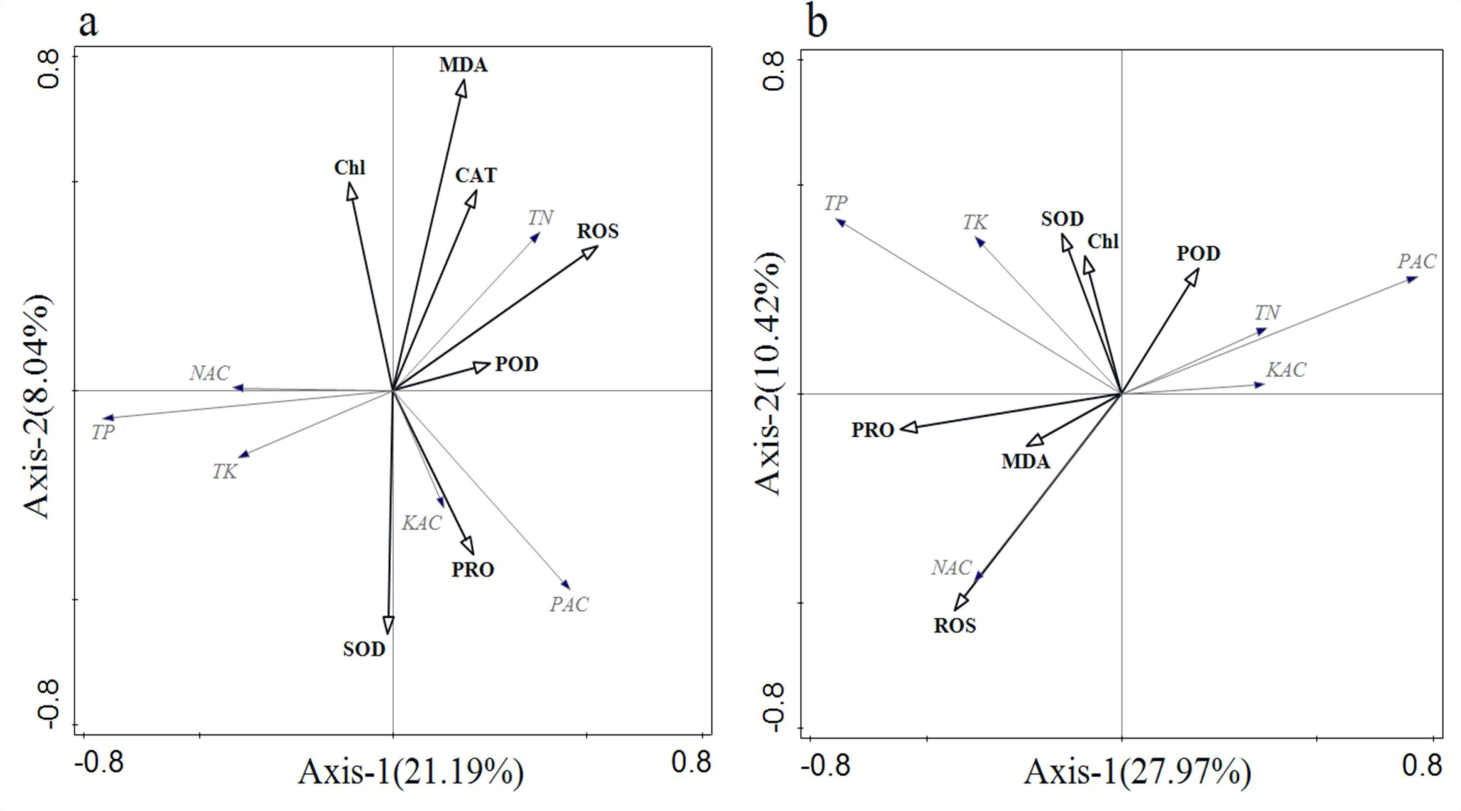
Redundancy analysis of soil physicochemical properties and plant physiological and biochemical indicators. **(A)** vegetative growth period; and **(B)** reproductive growth period.

In **Figure 7A**, the cumulative percentage of variance explained by the two ordination axes in the RDA result ranged from 29.23% to 38.39%. The ranking outcomes provide insights into the relationship between soil nutrients and rice enzyme activities. During the vegetative growth period, positive correlations were observed between PAC and PRO, as well as SOD, while negative correlations were noted between NAC and MDA, CAT, POD, and ROS. In contrast, during the reproductive growth period (**Figure 7B**), PRO, MDA, and ROS were negatively correlated with PAC and positively correlated with NAC.

## 4 Discussion

### 4.1 Effects of nutritional stress on the agronomic characteristics of rice

Nutritional stress exerts a significant influence on the agronomic traits of rice. The findings of this study reveal that plant height, number of effective panicles, 1000-grain weight, and yield were reduced under conditions of N deficiency, P deficiency, and K deficiency compared to full fertilization. In conditions of N deficiency, the plant height and number of effective panicles in rice were observed to be the lowest among all treatments, highlighting the crucial role of N in these agronomic traits. Considering all yield factors collectively, it is evident that N, P, and K positively influenced the vegetative growth of rice, aligning with the findings reported by Zhang (Zhang et al., 2022). Research findings indicate that as the quantities of N, P, and K applied in the fertilization regimen were decreased, there was a corresponding decrease in rice yield (Wu and Zhang, 2023). The experimental results indicate that fertilization has the potential to enhance rice yield. N and P were identified as the primary limiting factors for rice yield, with N exerting the most significant influence on yield-related factors such as plant height, effective panicles, and 1000-grain weight. The constraint on growth imposed by nutritional stress may stem from the inadequate acquisition of essential and well-balanced nutrients by the organism.

### 4.2 Effects of nutritional stress on the physical and chemical properties of soil and rice enzyme activity and antioxidant factors

Research findings suggest that the application of N fertilizer plays a crucial role in the vegetative growth and yield of rice (Djaman et al., 2016). As the rice plants developed, the activation coefficient of N progressively increased. The concurrent application of N and P enhanced the utilization efficiency of soil N. Similarly, the combined application of P and K improved the utilization efficiency of soil P and K. Numerous studies (Darmawan et al., 2009; Djaman et al., 2016) have demonstrated that P application can elevate the available P content in the soil, a finding that was corroborated by the results of the present study. P is a vital nutrient essential for crop metabolism, collaborating with N fertilizer to enhance rice growth. In this investigation, a highly significant negative correlation was observed between soil available P and ammonium N during the rice reproductive growth period, aligning with findings from prior research (Xiao et al., 2023). In cases of K deficiency, crops exhibit reduced absorption, underscoring the significance of K fertilizer application in augmenting the available K content in the soil (Nakamura et al., 2010). Consequently, fertilization enhances the soil NAC and KAC while diminishing the PAC.

With increasing stress intensity, there was an upward trend observed in POD activity. Specifically, during the rice vegetative growth period, the POD activity surpassed that of the full fertilization treatment in the K-deficient treatment, contrary to the findings reported by Zhu et al (Zhu et al., 2024). The analysis of CAT activity changes revealed a decreasing trend over time in the P deficiency treatment across the two periods, consistent with findings from prior studies (Huang et al., 2013; Hussain et al., 2017; Hussain et al., 2019). As a response mechanism to reactive oxygen stress, SOD exhibited a pattern similar to the changes in reactive oxygen concentration. There was no significant change observed between the two periods. However, under adverse stress conditions, SOD activity notably decreased (Wang et al., 2019). In the K deficiency treatment during the reproductive growth stage, there was a corresponding increase in SOD activity with the rise in ROS concentration, aligning with previous research findings. In an experiment involving foliar fertilizer application on rice, Peng Jiayuan (Peng et al., 2023) observed that the chlorophyll content of rice was elevated in the later growth stage, followed by a subsequent decrease until stabilization during the reproductive growth period. Stress was identified to exert an inhibitory effect on photosynthesis, a finding that is in line with the outcomes of the present study (Fariduddin et al., 2009).

In conclusion, CAT serves as the primary decomposer of H_2_O_2_ during the vegetative growth period, whereas POD plays a predominant role during the reproductive growth period. The alterations in SOD activity correspond with changes in reactive oxygen levels. These three enzymes collaborate to regulate the free radical content within the plant at a stable level, thereby preventing physiological and biochemical changes induced by free radicals. As the growth period advanced, N and P stress led to an accumulation of proline in the plants. The severity of membrane lipid peroxidation induced by N and K stress decreased, whereas N and P stress exacerbated membrane lipid peroxidation. To enhance the stress resistance of rice, it is recommended to reduce P fertilizer application and increase K fertilizer input during the reproductive growth period.

### 4.3 Relationship between soil nutrients and rice antioxidant enzymes

Soil nutrients have a direct impact on enzyme activities. In conditions of high N availability, plants can uptake a substantial amount, leading to an increase in proline and malondialdehyde contents in the plants. Concurrently, the activity of POD also increases accordingly. A study (Yan et al., 2021) has demonstrated that inadequate or excessive soil N supply resulted in reduced activities of SOD and POD, along with an accumulation of reactive oxygen species. Additionally, this condition led to elevated levels of the membrane lipid peroxidation product MDA and accelerated leaf senescence. In this experiment, POD, CAT, SOD, and MDA were all positively correlated with N, aligning with findings from a prior study (Meng, 2023). K, being a crucial constituent of plant enzymes, directly participates in substance synthesis. Elevated soil K content typically enhances plant enzyme activity. Nevertheless, in this experiment, the association between K and plant enzymes was not robust, contrary to the findings reported in previous studies (Meng, 2023). With prolonged stress exposure, PRO was notably influenced by K, while CAT was affected by P. The application of P and K could mitigate stress-induced damage and decrease the levels of oxidative substances. The relationship between plant photosynthesis, proline, and CAT suggests a synergistic effect, signifying that the efficient advancement of photosynthesis enhances enzyme activity. The potential explanation could be that organisms experiencing nutritional stress require extra energy and resources to address nutrient deficiencies, hindering their ability to efficiently mobilize defense mechanisms against other adverse factors. This weakened response severely impacts the plant’s survival and adaptation capabilities. Furthermore, under nutritional stress conditions, plants may enhance their capacity to uptake and transport specific nutrients. They can also improve their efficiency in acquiring limited nutrients from the soil by modifying their root structure and physiological functions in response to nutrient deficiencies.

## 5 Conclusion

In this study, rice plants subjected to N, P, and K deficiencies were selected as the experimental subjects. Antioxidant enzyme activities and soil physicochemical properties of the rice plants were assessed during both the vegetative growth and reproductive growth stages.

The research findings demonstrated that the full fertilization treatment markedly enhanced rice yield, as well as the NAC and KAC in the soil. Plant growth was constrained under nutrient stress conditions. The elevation of NAC was associated with the stimulation of PRO activity, MDA levels, and ROS content during the vegetative growth stage, in contrast to the results observed during the reproductive growth stage. During the later stages of plant growth, K emerges as a primary factor influencing the antioxidant enzyme system. Therefore, enhancing the input of K is recommended to enhance the plant’s stress resistance.

Understanding the physiological response mechanisms of plants under nutritional stress and the principles governing crop nutrition requirements enables the development of more scientifically informed and rational fertilization strategies. This, in turn, can enhance fertilizer utilization efficiency and minimize wastage.

## Data Availability

The raw data supporting the conclusions of this article will be made available by the authors, without undue reservation.
